# Separation of Chromatographic Co-Eluted Compounds by Clustering and by Functional Data Analysis

**DOI:** 10.3390/metabo11040214

**Published:** 2021-03-31

**Authors:** Aneta Sawikowska, Anna Piasecka, Piotr Kachlicki, Paweł Krajewski

**Affiliations:** 1 Department of Mathematical and Statistical Methods, Poznań University of Life Sciences, Wojska Polskiego 28, 60-637 Poznań, Poland; 2 Institute of Bioorganic Chemistry, Polish Academy of Sciences, Z. Noskowskiego 12/14, 61-704 Poznań, Poland; apiasecka@ibch.poznan.pl; 3 Institute of Plant Genetics, Polish Academy of Sciences, Strzeszyńska 34, 60-479 Poznań, Poland; pkac@igr.poznan.pl (P.K.); pkra@igr.poznan.pl (P.K.)

**Keywords:** chromatographic peak separation, chemometrics of chromatographic data, computational peak deconvolution, functional principal component analysis, simulation, metabolomics

## Abstract

Peak overlapping is a common problem in chromatography, mainly in the case of complex biological mixtures, i.e., metabolites. Due to the existence of the phenomenon of co-elution of different compounds with similar chromatographic properties, peak separation becomes challenging. In this paper, two computational methods of separating peaks, applied, for the first time, to large chromatographic datasets, are described, compared, and experimentally validated. The methods lead from raw observations to data that can form inputs for statistical analysis. First, in both methods, data are normalized by the mass of sample, the baseline is removed, retention time alignment is conducted, and detection of peaks is performed. Then, in the first method, clustering is used to separate overlapping peaks, whereas in the second method, functional principal component analysis (FPCA) is applied for the same purpose. Simulated data and experimental results are used as examples to present both methods and to compare them. Real data were obtained in a study of metabolomic changes in barley (*Hordeum vulgare*) leaves under drought stress. The results suggest that both methods are suitable for separation of overlapping peaks, but the additional advantage of the FPCA is the possibility to assess the variability of individual compounds present within the same peaks of different chromatograms.

## 1. Introduction

The systems biology approach requires large-scale experiments in which multiple genetically polymorphic biosources are studied under varying environmental conditions. The number of the studied genotypes may be very large, especially in plant or animal genetic studies, in which large populations of forms created by artificial breeding or collected in nature are used. In plant research, where the creation of large experimental populations is relatively easy, repeated measurements are also performed at several time points of the performed experiment to deepen the understanding of plant metabolism. Although analytical techniques have become increasingly precise and the number of technical measurement replications has gradually decreased, the number of biological replications must be sufficient to properly estimate the natural variation. All of this leads to the situation in which the experiments performed have a multifactorial structure and the number of analyzed samples is very large. Moreover, modern chromatography uses new high-throughput and more sensitive instruments and protocols that generate a large amount of data. Although many methods of data analysis have been proposed in the literature, for example, for metabolomic data [1,2,3,4], the problem of separating co-eluting compounds in large chromatographic datasets has not, so far, been satisfactorily solved.

Chromatographic co-elution occurs when two or more compounds do not chromatographically separate. This can be handled by increasing the selectivity, by improving the efficiency of the chromatography, by changing the chemistry of the mobile phase, stationary phase, temperature, or column length, or by using more than one column in a line [5]. However, the total separation of all compounds by chemical and technical solutions is often difficult or impossible to achieve within a reasonable time for a large experiment with biological samples, and it would highly increase the cost of analysis.

In this situation, numerical peak separation can be an effective strategy, especially for a large number of samples. For peak deconvolution, functional representation of chromatographic peaks can be used. The quality of data analysis using such representations depends on the shape of peaks [6]. The exponentially modified Gaussian (EMG) function is the most popular function used in peak deconvolution methods [7,8,9]. The parabolic Lorentzian-modified Gaussian (PLMG) model was applied in [10] to the deconvolution of peaks in binary mixtures of structurally related compounds that are highly overlapping. Using the Gaussian distribution function (GD), the Lorentzian function, and the EMG function, three deconvolution methods were tested [11] for the high-performance liquid chromatography (HPLC) analysis of sugars in samples of kiwi juice. It was revealed that the EMG function was the best to describe overlapping chromatographic peaks. Therefore, this method was used for the analysis of kiwi juice samples. In [12], an EMG peak deconvolution routine was constructed for chromatography using a combination of two EMG formulas and linear optimization methods for stand-alone overloaded peaks. Separation algorithms were developed for liquid chromatography coupled with a photodiode array detector in [13,14]. In the first publication, a method based on a model of generalized Gaussian reference curve measurement (GGRCM) and an algorithm of multi-target intermittent particle swarm optimization (MIPSO) was proposed. In the second paper, an algorithm that uses a bidirectional EMG function and then separates peaks using multivariate curve resolution-alternating least squares (MCR-ALS) was presented to estimate chromatograms. Several algorithms for peak deconvolution by non-linear regression were presented [15]. The polynomially modified Gaussian (PMG) model was used to describe peaks. In [6,16], methods for locating the boundaries of chromatographic peaks were given based on the Gaussian peak model, PLMG, and models used in the PeakFit program (Systat Software, Inc., San Jose, CA, USA), which is commonly used to fit peak models to experimental data. The linearly modified Gaussian (LMG) model was proposed in the description of peaks in liquid chromatography [17]. A procedure for resolving noisy overlapped peaks in DNA separations by capillary electrophoresis (CE) was developed [18]. The method based on wavelet transforms has a better performance for signals with a significant quantity of noise than the commercial software PeakFit. Application of recursive peak detection based on continuous wavelet transforms was proposed [19] to resolve the overlapping peaks detected in chromatographic signals. That method also gives more accurate positions of peaks and smaller relative fitting errors than PeakFit does. However, methods using those models and methods with automatic peak detection focus only on peak separation from one chromatogram, not taking into account many chromatograms in large data with multifactorial structures. The goal of the methods presented in this paper is to perform peak separation to compare them and statistically analyze peaks in all chromatograms, not only in individual ones. Previous methods cannot be applied in this situation and their applications are not described for such cases.

Chromatographic techniques with ultraviolet (UV) and fluorescence detectors are widely used in the determination of primary and secondary metabolites. These methods are precise and sensitive, and sample preparation prior to this analysis demands usually only a simple extraction step. Quantitative analysis based on chromatographic peak measurement is a well-established, fast, and accurate method in studies on agronomically and industrially important metabolites such as carotenoids [20], chlorophylls [21], phenolics [22], flavonoids [23], and betalains [24]. HPLC-UV methods are customarily used for mycotoxins’ determination in foods [25,26] and other biological samples [27]. They are also useful for screening metabolites’ profiles when studying biosynthetic pathways and metabolites’ functions (for example, biosynthesis of tryptophan derivatives in the immune system of *Arabidopsis thaliana*, as was described [28]). Therefore, in many cases, when liquid chromatography (LC) is used alone (without mass spectrometry) and some compounds co-elute, their separation is essential for quantitative and qualitative analyses.

In this paper, we compare two methods of peak separation in chromatograms recorded with light absorbance or fluorescence detectors and obtained by separation techniques such as LC or capillary electrophoresis. Both methods are applied for the first time to the considered problem in such chromatographic data. The first method of peak separation considered in this paper is based on clustering, and the second method uses functional principal component analysis (FPCA). Clustering separates chromatographic peaks by dividing convolved fragments of chromatograms into groups consisting of similar peaks with respect to their shape. FPCA does not separate peaks explicitly, but by detecting sub-peaks with the greatest variability, it provides an optimal, possibly multidimensional, peak representation. None of the methods presented in the literature focus on better preserving differences between experimental variants, which is crucial for statistical analysis. The application of FPCA to chromatographic data presented in this paper gives that additional advantage. Therefore, when considering a comparison between samples, peaks with different areas are highlighted, which is consistent with the main aim of comparative untargeted metabolomics. We present both methods and compare their performance using examples of real and simulated metabolomic data, concentrating not on chemical interpretation but on methodological aspects.

## 2. Results

### 2.1. Simulations

**Figure 1:**
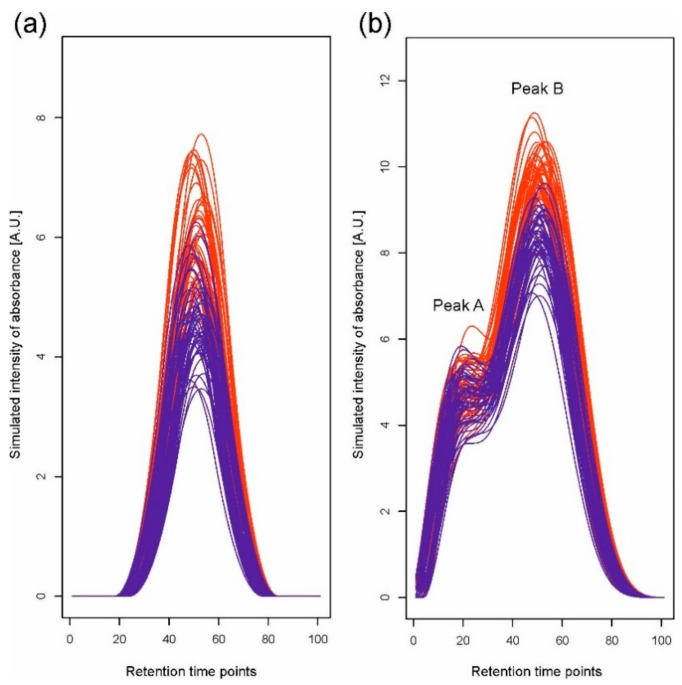
One of the simulated datasets for (**a**) a single peak and (**b**) a double peak, which consists of two overlapping peaks, A and B. Different colors indicate different experimental variants.

The scenario of simulations corresponds to a situation in which it is required to compare two experimental variants (e.g., groups of patients, plant genotypes, and treatments), each represented by a number of chromatograms corresponding to individual samples (replications). We simulated data for two situations: first, when a single peak representing one compound is observed, and second, when a peak representing a mixture of two compounds, i.e., a double peak, is observed. For the single peak, we assume that the mean compound concentration differs between the two experimental variants; for the double peak, we assume that the mean concentrations differ only for one of the mixed compounds. Simulated datasets were constructed for two variants, each represented by 50 chromatograms based on 100 retention time points. We used 6 B-spline functions [29,30] of order 3 to generate chromatograms. We created linear combinations of basis functions with the coefficients drawn from normal distributions *N*(*μ*, 1) to achieve simulation of a single peak (Figure 1a) and a double peak (Figure 1b). For the single peak, the coefficient for the fourth basis function was nonzero, and for the first variant, its mean *μ* was equal to 8. For the second variant, we changed the concentration level by taking *μ* = 6, 7, 7.25, 7.5, 7.75, and 8. For the double peak, to simulate a convolution of two peaks, nonzero coefficients for only the third and fifth basis functions were generated. Using two B-splines, we created overlapping peaks, A and B. The means of the simulated concentration for both variants in peak A were equal to eight, while in peak B, the means for variants differed and were equal to 16 for the first variant and 14, 15, 15.25, 15.5, 15.75, and 16 for the second. In all situations, for each combination of two mean values for two variants, 1000 datasets were generated. We also assumed random retention time shifts by −3 to 3 time points in simulations in addition to random changes of chromatogram levels. The step of peak detection was performed without smoothing of the second derivative since our simulated data are not noisy.

Each generated dataset was analyzed using Methods 1 and 2. Using hierarchical clustering (with 1000 bootstrap samples) in Method 1, the simulated chromatograms within detected peaks were initially grouped into 1, 2, …, 18 clusters depending on the variant and dataset, but the algorithm applied for joining peaks from different clusters finally allowed to define one peak for a single peak situation and two peaks for a double peak situation.

In Method 2, we assumed that a standard peak consists of about 60 retention time points (which results from the practice) and can be a convolution of six components; we used the set of six basis functions comprising one B-spline of order 3 per 10 retention time points. FPCA was carried out with the number of principal components equal to five, which is related to a mixture of six compounds in one peak. Note that this assumption is not restrictive, and if a peak is a convolution of two components, the method will show two components with a high level of total variance. Therefore, this assumption does not limit the number of compounds shown in one peak.

**Figure 2:**
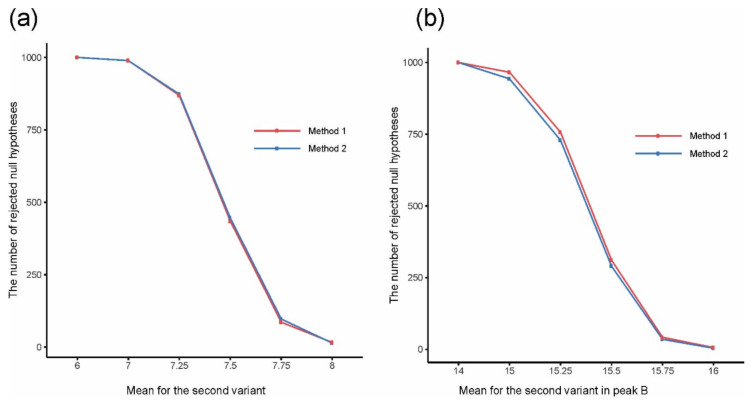
The number of rejected null hypotheses about equality of mean values for two experimental variants for (**a**) a single peak and (**b**) a double peak, depending on the mean value for the second variant in (**a**) a single peak and (**b**) peak B.

The analysis of the simulated data showed that both methods gave the expected number of separated peaks and both were appropriate for solving the problem of convolution. We statistically tested the significance of differences between variants and compared the numbers of rejections of the null hypothesis of variant equality by the two methods in series of 1000 simulations to discover which method is better for detecting differences between variants (Figure 2). In the case of a single peak, the *t*-test was applied for testing H_0_: *μ*_1_ = *μ*_2_, H_1_: *μ*_1_ ≠ *μ*_2_, where *μ*_1_ and *μ*_2_ are means for variants 1 and 2, respectively. In the case of a double peak, Hotelling’s *T*^2^-test was applied to test the corresponding bivariate null hypothesis. Calculations were performed on the values of integrated peaks using clustering for Method 1, and on the first one or two FPCA scores (depending on the considered peak, if it was a single or a double peak) for Method 2 based on FPCA. In the case of a single peak, both methods gave very similar results (Figure 2a). In the case of a double peak, FPCA gave slightly better results than clustering, so FPCA better kept differences between variants (Figure 2b).

### 2.2. Real Data Analysis

**Figure 3:**
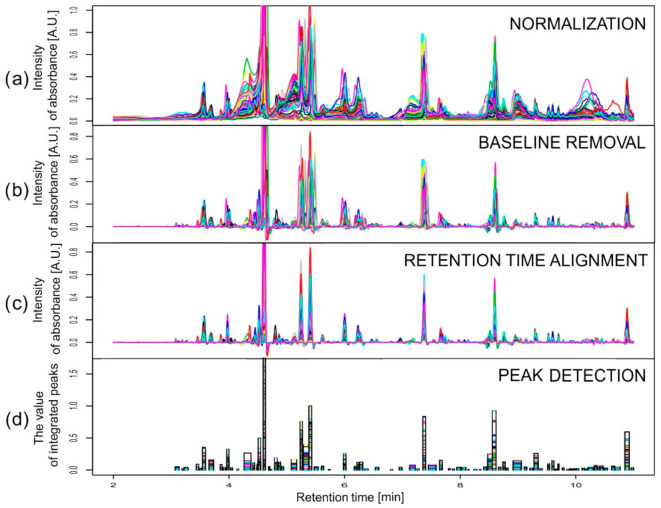
Pre-processing data from chromatograms recorded at 330 nm for Maresi after the following steps: (**a**) normalization by sample mass, (**b**) differentiation, (**c**) correlation optimized warping (COW), and (**d**) peak detection. The last plot shows the width of peaks and their value after integration for individual chromatograms.

Data obtained in an experiment on barley were baseline corrected using the rolling ball method with two parameters: width of the local window for minimization/maximization set to 50 and width of the local window for smoothing also set to 50. At the alignment stage, a stepwise choice of the reference chromatograms was conducted separately for each wavelength (280 and 330 nm). The reference chromatogram selection was made first inside the varieties and then between them, according to values of the similarity index. For both wavelengths considered, the chromatograms obtained for the third replication of variety Stratus on the third day of the drought variant II were chosen as the common reference. Retention time alignment and peak detection were performed according to the description above and [31], but with different warping parameters for correlation optimized warping (COW): the segment length *m* ∈ {180, 216, 270, 360, 540} and the slack parameter *t* ∈ {1, 3, 5, 8, 10, 12}. These values were chosen according to the observed peak widths and shifts of the chromatograms, applying the rule of thumb. Moreover, *m* had to be selected as a divisor of the chromatographic profile length (10,801) minus one. The best combinations in a grid of 30 points for segment lengths and the slack parameters were found independently for each variety. The peak factor, the simplicity, and their sum, called the warping effect [32], were used to describe the efficiency of the alignment. The obtained peak factor values were very high (close to 1) for all varieties and both wavelengths, which means that there were very small changes in the peak areas and shapes caused by the warping. The values of simplicity varied from about 0.3 for Cam/B1/CI (λ = 280 nm) to about 0.9 for Harmal (λ = 280 nm), so the differences between chromatograms obtained for Cam/B1/CI for different drought treatments and time points were large in contrast to those for Harmal. The simplicity values for all varieties and for all parameters were smaller for chromatograms recorded at 280 nm than for those recorded at 330 nm. The peak detection in the aligned chromatograms revealed 84 and 83 peaks for wavelengths of 280 and 330 nm, respectively. Figure 3 presents an example of the results of all the pre-processing steps for chromatograms recorded at 330 nm for the variety Maresi. Two peaks were chosen as interesting examples among all 167 peaks to show the problem of compounds eluting at very similar retention times and to compare two methods of dealing with the problem. Data from different wavelengths are treated separately. At one wavelength, there can be deconvolution method needed at the same retention time as in another one in which that problem does not occur. Integration of two wavelengths is the last step before statistical data analysis and it is another problem that is not crucial and not considered in this publication (for more, see [6]). Metabolites were identified by mass spectrometry–liquid chromatography as well as nuclear magnetic resonance; for details, see [33,34].

#### 2.2.1. Example 1

**Figure 4:**
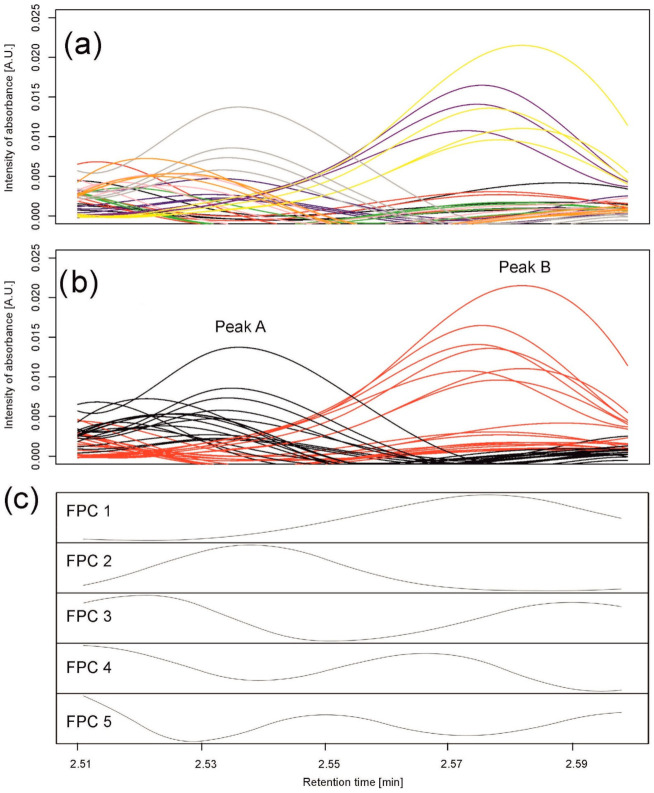
Data analysis for peaks observed in barley samples taken on the 6th day of the drought variant I for wavelength 280 nm. (**a**) Observed chromatograms; colors indicate varieties: black—Maresi; red—Lubuski; blue—Georgia; green—Harmal: pink—Cam/B1/CI; purple—MDingo; yellow—Morex; gray—Sebastian; and orange—Stratus. (**b**) Observed chromatograms; colors indicate two clusters obtained by Method 1. (**c**) First five eigenfunctions obtained by Method 2 accounting for 99.93% of the total variance. Functional principal component 1 (FPC1) (76.88% of the variance) corresponds to peak (cluster) B, and the component FPC2 (18.32% of the variance) to peak (cluster) A.

In the first example, we selected 35 chromatograms from the whole set of data for all varieties observed on the sixth day of the drought variant I for wavelength 280 nm and retention times from 2.51 to 2.6 min (112 retention time points); see Figure 4a. The selection was carried out to show the problem of elution of different compounds at very similar retention times but present in different chromatograms that are analyzed together.

##### Method 1

Using hierarchical clustering (with 1000 bootstrap samples), pre-processed chromatograms within this peak were grouped into two clusters of 18 and 17 chromatograms; see Figure 4b. Peak detection in each cluster resulted in two peaks—one peak per group—peak A from 2.510 to 2.546 min and peak B from 2.553 to 2.598 min. The first group comprised all samples for three varieties (Georgia, Sebastian, and Stratus) and mixtures of the others; the second group comprised all samples for two varieties (Maresi and Morex) and mixtures of the others. The chromatograms were well grouped, separating two different peaks; see Figure 4b.

##### Method 2

Similar to the simulations, we assumed that a standard peak consists of about 60 retention time points and can be a convolution of six peaks. Therefore, the number of basis functions was 10, with one B-spline (of order 3) per 10 retention time points. A functional principal component analysis was performed with the number of principal components equal to five; see Figure 4c. By observing the maxima of the first two functional principal components (FPCs) (eigenfunctions) FPC1 and FPC2, explaining, together, 99.93% of variation between the chromatograms, we clearly see that they correspond to peak B and peak A found by clustering, respectively.

##### Experimental Validation

**Figure 5:**
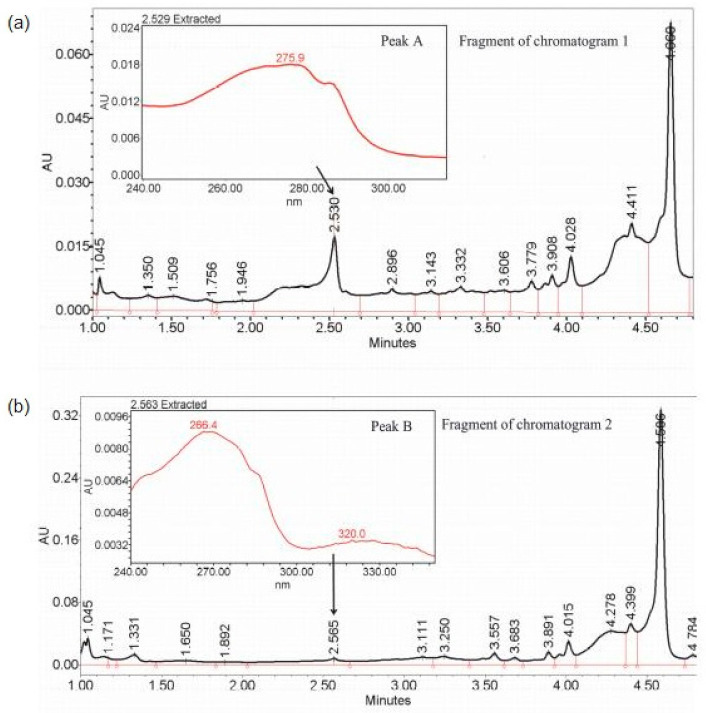
Fragment of: (**a**) chromatogram 1 and (**b**) chromatogram 2, belonging to different clusters, with peaks A and B (also shown in Figure 4b) corresponding to retention times of 2.530 and 2.564 min, respectively, and with highlighted maxima of absorption λ_max_ = 275.9 nm in chromatogram 1 and λ_max_ = 266.4 and 320 nm in chromatogram 2, indicating two different metabolites.

Peak identification was performed as described in the Supplementary Materials: Instrumental analysis. Figure 5 shows two chromatograms—1 and 2—belonging to different clusters, with peaks A and B corresponding to retention times of 2.530 and 2.564 min, respectively, which is in the range of detected peaks. A small difference between the two peaks initially resulted in their recognition as a single peak from 2.501 to 2.603 min. However, both separating methods allowed the two peaks to be distinguished, and, indeed, the analysis of the raw data showed that the compounds present in peaks A and B had different maxima of absorption. In chromatogram 1, peak A had one maximum at λ_max_ = 275.9 nm, whereas in chromatogram 2, peak B was characterized by two local maxima at λ_max_ equal to 266.4 and 320.0 nm, which indicates the existence of two metabolites having similar physicochemical properties but belonging to different classes of chemicals. Moreover, the instrumental analysis confirmed the grouping in Method 1 by indicating which of peaks A and B was in which sample. Therefore, it was concluded that there was no metabolite represented by peak A in the varieties Maresi and Morex and no metabolite represented by peak B in the varieties Georgia, Sebastian, and Stratus.

**Figure 6:**
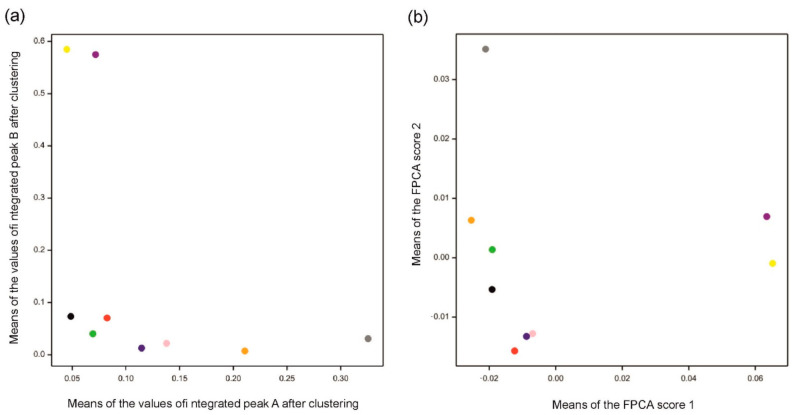
Means for varieties obtained by (**a**) Method 1 using clustering and (**b**) Method 2 using functional principal component analysis (FPCA). Mean values in (**a**) were computed over four replications for each variety over integrated peaks A and B obtained by clustering. Mean values in (**b**) were computed over four replications for each variety for the two first FPC scores. Different colors indicate varieties (see caption of Figure 4). Note that positions of varieties along FPC score 1 correspond to those along the values of peak B and positions along FPC score 2 correspond to those for peak A.

The results for both methods are consistent with the data (see Figure 6). Compared to Figure 4a,b, indeed, chromatograms denoted by a “gray” spot are mostly separated from others in peak A, while “yellow” and “purple” are the most different from others in peak B. Functional principal component 1 corresponds to peak B and functional principal component 2 corresponds to peak A. Moreover, the Pearson correlation coefficient between integrated values over peak A and values of FPC2 is 0.87, while the correlation is 0.99 for peak B and values of FPC1. Figure 6a visualizes differences and similarities among varieties with respect to integrated peaks A and B, while Figure 6b focuses on the whole profiles of chromatograms summarized by the first two functional principal components.

#### 2.2.2. Example 2

**Figure 7:**
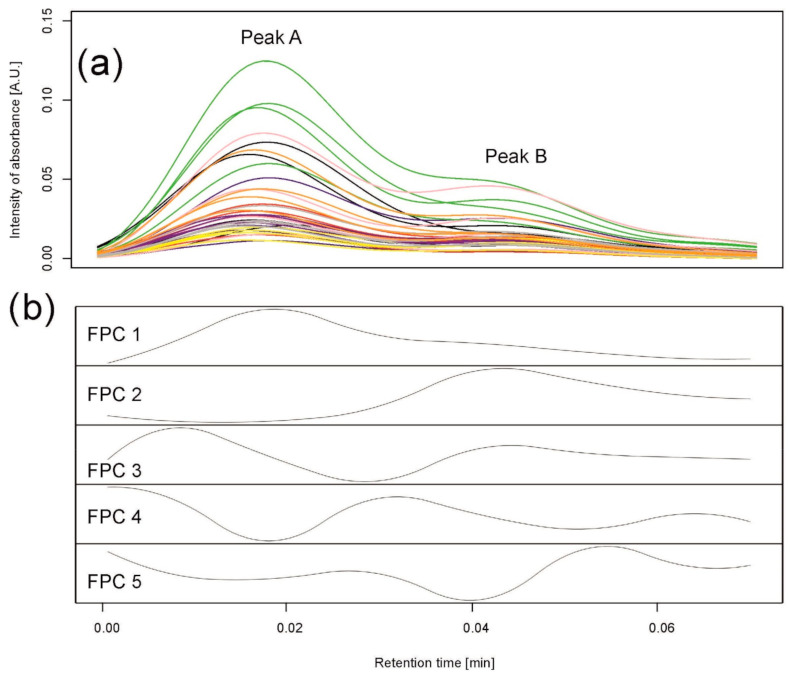
Data analysis for peak observed on the 6th day of control for wavelength 330 nm. (**a**) Observed chromatograms. Different colors indicate varieties: black—Maresi; red—Lubuski; blue—Georgia; green—Harmal; pink—Cam/B1/CI; purple—MDingo; yellow—Morex; gray—Sebastian; and orange—Stratus. (**b**) First five eigenfunctions accounting for 99.98% of the total variance. Component FPC1 (97.59% of the variance) corresponds to peak A, and component FPC2 (1.77% of the variance) to peak B.

In the second example, we selected 36 chromatograms from the whole set of data for all varieties observed on the sixth day of control for wavelength 330 nm and retention times from 9.27 to 9.35 min (89 retention time points); see Figure 7a. This situation illustrates the problem of overlapping peaks occurring together in each single chromatogram.

##### Method 1

Using hierarchical clustering (with 1000 bootstrap samples), pre-processed chromatograms within this peak were grouped into only one cluster. In this instance, it is impossible to achieve clusters indicating two different peaks since all chromatograms have a similar shape.

##### Method 2

FPCA was performed in the same way as in Example 1, with the number of principal components equal to five; see Figure 7b. FPC1 corresponded to peak A and FPC2 to peak B.

##### Experimental Validation

**Figure 8:**
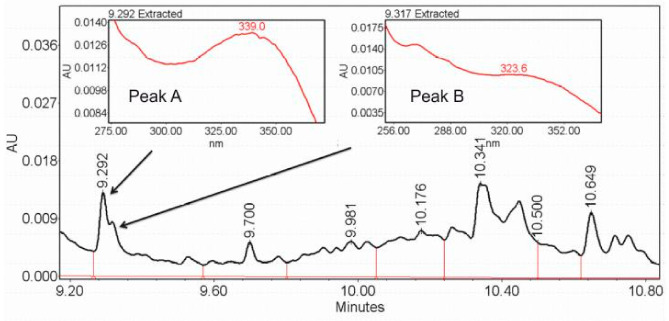
Fragments of the chromatogram from Example 2 with two overlapping peaks corresponding to retention times of 9.293 and 9.320 min and with highlighted maxima of absorption λ_max_ = 339.0 nm for peak A and λ_max_ = 323.6 nm for peak B, indicating two different metabolites.

Peak identification was performed as described in the Supplementary Materials: Instrumental analysis. Figure 8 shows one of chromatograms from Example 2, with two overlapping peaks corresponding to retention times of 9.293 and 9.320 min, respectively, which is in the range of detected peaks. A small difference between two peaks resulted in their recognition as a single peak from 9.27 to 9.35 min. However, the analysis of the raw data showed that peaks A and B had different maxima of absorption: λ_max_ = 339.0 nm for peak A and λ_max_ = 323.6 nm for peak B, which indicates the existence of two metabolites.

## 3. Discussion

The main aim of this paper was to define and compare two methods of chromatographic data processing aimed at separating overlapping peaks representing co-eluting chemical compounds in the situation where chromatography is used as the sole analytical method, not followed by mass spectrometry. Our proposed processing flowchart cannot be applied for compound separation of LC-MS data due to its three-dimensional form with *m/z* value. However, it can be adapted to a total ion chromatogram from LC-MS, which is two-dimensional with retention time and intensity, as in LC used alone, which is considered here. We described the comprehensive procedure for utilizing LC-MS metabolomic raw data to draw biological conclusions in [35]. However, mass spectrometry analysis requires extensive knowledge and experience. Moreover, the cost of apparatus is often a limiting factor. Therefore, many analytical applications can be adapted to use chromatography alone, which simplifies the analysis and reduces its cost. We are aware that chromatographic data analysis is a multi-step process, in which all stages contribute to the final result. Simplification of processing methods and intuitive useful software are still matters of the future. 

Before the application of the considered methods of separating peaks, first—in the case of real data—pre-processing was conducted. Among many alignment methods [36,37,38], dynamic time warping is the most commonly used, but since it is sensitive to different peak intensities and might lead to unsatisfactory alignment [39], COW [32,36,37,39,40] was chosen. Alignment by COW does not require prior chromatographic peak detection, in contrast to several other methods [41,42,43,44]. Some parameters in alignment were optimized automatically, for example, the UHPLC-UV data warping segment length and slack value; for them, the size of the dataset was a factor limiting, to some extent, the optimization process. Others, such as the UHPLC-UV peak detection thresholds, were tried with different values, and several rounds of consultations on the number and quality of detected metabolites were conducted with the experimenters. Close collaboration with the analytical chemists allowed for improvement of metabolite identification using mass spectrometry and, in some cases, nuclear magnetic resonance spectroscopy [33,34]. 

The method of separating peaks by clustering is the approach that allows the establishment of groups of chromatograms possessing different patterns of signals. In the case of real data with different peaks at the same time in different profiles, chromatograms were well grouped, thus separating peaks. However, in the case of overlapping peaks in each profile with a similar shape, clustering was not able to create groups corresponding to their different components. If clustering could indicate some groups, they would be formed because of differences in intensities or because of small retention time shifts, which is not interesting and meaningful. FPCA gave equally satisfactory results for both cases. Both deconvolution methods allowed the two peaks to be distinguished in the case of Example 1 of real data, and, indeed, the analysis of the raw data showed that peaks A and B had different maxima of absorption. The existence of two different metabolites was also proven by mass spectrometry in the case of Example 2 of the real data experiment, but here, a consistent answer was given only by FPCA. The FPCA method gives many variables, and the number variables used, that is, of functional principal components, can be limited by the threshold on the explained variance. Each peak can be interpreted as a convolution of two peaks in this particular case, so for this method, the results can always be presented in a two-dimensional coordinate system (Figure 6). Such a presentation of results is not always possible for a method using clustering (Example 2).

## 4. Materials and Methods

### 4.1. Motivating Data

Our motivating example came from an investigation of the effects of water shortage on the levels of secondary metabolites in varieties of barley, measured repeatedly during a drought application period (performed as a pilot study for a larger systems biology project) [31]. For 9 varieties (Georgia, Maresi, Lubuski, Sebastian, Stratus, Morex, Cam/B1/CI, Harmal, and MDingo) 3 different drought treatments (I, II, I+II), and a control, data were obtained at 8 time points and in 4 biological replications. More details of the experimental setup and the biological insight were published previously [33]. In this three-factor experiment, the factors were as follows: variety, drought variant, and time point during drought. The metabolite level measurements were performed using an ultra-high performance liquid chromatograph with a photodiode array detector (UPLC-PDA; Acquity with eλ diode array detector, Waters, Milford, MA, USA). The total number of samples subjected to metabolite profiling was 547, with the number of retention time points exceeding 10,000; therefore, the number of observations was very large. As an example, two UV wavelengths (280 and 330 nm, characteristic for phenolic compounds) were selected. We chose this for simplicity of presentation, and although all wavelengths can be considered, in practice, scientists only investigate wavelengths representative for the analyzed compounds. The same data pre-processing was performed for both methods presented in this paper. The most time-consuming operation, the retention time alignment of the chromatograms obtained for different samples, had to be organized in an optimal way for our situation—it turned out that this could be achieved by application of the correlation optimized warping algorithm (COW) [36] with a specially chosen sequence of the reference chromatogram selections. For the same reasons, the selected peak detection method had to be applicable to a large set of chromatograms and integrated with the alignment.

The problem of overlapping peaks occurred after the peak detection because of the presence of metabolites with very similar retention time intervals. We also realized that in a large experiment such as this, with heterogeneous plant material, different metabolites with relatively similar retention times might be selectively detected in different samples (above the detection threshold). The existing deconvolution algorithms [15,45] that have been established to separate signals for co-eluting compounds could not be applied in our situation due to the large number of samples.

### 4.2. Data Pre-Processing

Pre-processing had to be performed for real data before the peak separation. Simulated data (described in Section 2.1) were constructed to imitate chromatograms obtained after the step of pre-processing. Here, we consider a chromatographic dataset in the form of a (retention time × sample) table processed in the following way [31]:

(a) Normalization: the raw data were divided by the mass of the appropriate extracted leaf sample.
(b) Baseline removal: chromatographic baselines were removed using the Rolling Ball algorithm based on [46] using package baseline in R.
(c) Retention time alignment: the algorithm of COW [36] was applied with the choice of reference chromatograms based on the maximum value of the similarity index.
(d) Peak detection: peaks, interpreted as the retention time intervals in which some types of metabolites or a group of metabolites with similar chromatographic properties occur, were detected in each single chromatogram using the second derivative smoothed by a cubic smoothing spline with the function smooth.spline in R [31]. The inflection points were located to find boundaries for the individual peaks, and peaks common for several (or all) chromatograms were built to compare peak areas between samples. At this stage, peaks that result from mixtures of compounds eluting at similar retention times appear, and these peaks should be subjected to separation.

### 4.3. Method 1: Peak Separation by Clustering

The problem of overlapping peaks can be solved by clustering after peak detection. In each of the detected peaks common for all chromatograms, we assume that there can be a mixture of compounds. If these compounds occur in different samples at different concentrations, the set of chromatographic profiles observed within the considered retention time interval can be divided into subsets (clusters) of relatively similar profiles. In our algorithms, clustering was performed using the hierarchical average link algorithm with a correlation-based similarity coefficient. We used numerical implementation of the method in the pvclust function in R, which has an additional advantage as it allows for automatic determination of clusters via bootstrap resampling. First, all chromatograms characterized with the presence of a peak in the considered retention time range were grouped. Then, for each group of chromatograms, step d) was performed. Obviously, in different obtained groups of chromatograms, there can be peaks with overlapping retention times and peaks including other peaks can also exist. Therefore, peak assignment between clusters was performed as follows.

First, for each peak *P* from one of the clusters and each *P*′ belonging to any other cluster, we calculated *the percentage of overlap* as:

*percentage of overlap* (*P*,*P*′) = 100 × *length*(*P*∩*P*′)/*min*(*length*(*P*),*length*(*P*′)). (1)

Then, we constructed a list of candidate peaks for joining by listing the pairs for which the percentage of overlap is greater than 80%. Let the number of overlaps for peak *P* be the number of peaks that overlap with it by more than 80%. The one with the greatest number of overlaps is selected among all such peaks. Then, the one with the greatest number of overlap*s* is again selected among all peaks that are candidates for joining with the selected peak. The process is repeated until peak *P* is assigned to the peak *R* with the greatest number of overlaps among all peaks considered during this stage of searching. The result of the iteration is that peak *R* is a peak inclusive of a large part of peak *P* (not necessarily more than 80%) and the greatest number of the large parts of other peaks. However, different metabolites with very similar retention times might be separated even if their retention times overlap with other metabolite retention times less than 80%.

Let us consider an example. Assume that the following list of candidates for joining (overlapping by more than 80%) was constructed for 10 peaks from different clusters but corresponding to the same metabolite:

peak 1 to 2, 3, 4 with *percentage of overlap* accordingly (90%, 85%, 81%);
peak 2 to 1, 9 with *percentage of overlap* accordingly (90%, 82%);
peak 3 to 1 with *percentage of overlap* of 85%;
peak 4 to 1, 5, 8, 9 with *percentage of overlap* accordingly (81%, 82%, 84%, 90%);
peak 5 to 4, 8 with *percentage of overlap* accordingly (82%, 85%);
peak 6 to 8 with *percentage of overlap* of 90%;
peak 7 to 8 with *percentage of overlap* of 91%;
peak 8 to 4, 5, 6, 7, 10 with *percentage of overlap* accordingly (81%, 85%, 90%, 91%, 87%);
peak 9 to 2, 4 with *percentage of overlap* accordingly (82%, 90%);
peak 10 to 8 with *percentage of overlap* of 87%.

Let us consider peak 1. First, peak 4 is selected due to the greatest number of overlaps equal to four (among peaks 2, 3, and 4); then, for peak 4, peak 8 is selected with the number of overlaps equal to five (among peaks 1, 5, 8, and 9). The selection stops here because none of the candidates for peak 8 have more overlaps than five. Observe that the percentage of overlap between peaks 1 and 8 is smaller than or equal to 80%, but the percentage of overlap between peaks (1, 4) and peaks (4, 8) is greater than 80%. Therefore, peak 8 includes a large part of peak 1. Moreover, it includes the greatest number of the large parts of other peaks. It can be examined that in this example, all peaks are assigned to peak 8, which indicates one metabolite.

### 4.4. Method 2: Peak Separation by Functional Principal Component Analysis

Functional data analysis (FDA) is a branch of statistics that develops methods for the description of observations that can be assumed to come from realization of a function [29,30]. One of the FDA methods is FPCA, which we use as an alternative approach to peak separation. We briefly review the foundations of this method in the following.

The best way to see the idea of FPCA is to present it as a continuous alternative to the classical, multivariate principal component analysis (PCA). In PCA, for a set of *n P*-dimensional observations $x_{i}\;=\;\left(x_{i1},\;x_{i2},\cdots ,x_{iP}\right)$, i = 1, 2,⋯,n, of *P* random variables, we construct a set of *J*-dimensional principal component (PC) scores $y_{i}\;=\;\left(y_{i1},\;y_{i2},\cdots ,y_{iJ}\right)$, i = 1, 2,⋯,n, where $y_{ij}\;=\;\sum_{p\;=\;1}^{P}\;\xi_{jp\;}x_{ip}$. Coefficients ξ*_jp_* are chosen in such a way that the new variables, PC scores, are uncorrelated and their variances’ decrease so that a low-dimensional, adequate representation of data can be achieved by selecting the first few principal components. Numerically, PCA is performed by eigen analysis of the sample variance–covariance matrix.

In FDA, we assume that each observation is of the form $x_{i}\;=\;x_{i}\left(t\right)$, where the values of *t* come from an interval *T* of time or any other continuous space. In this space, we consider inner products of the form $\int_{T}^{}\xi \left(t\right)\;x_{i}\left(t\right)dt$ instead of linear combinations. The observations $x_{i}$, i = 1, 2,⋯,n, can be represented by a set of *J*-dimensional scores $y_{i}\;=\;\left(y_{i1},\;y_{i2},\cdots ,y_{iJ}\right)$, where $y_{ij}\;=\;\int_{T}^{}\xi_{j}\left(t\right)\;x_{i}\left(t\right)dt$, where the functions (“eigenfunctions”) $\xi_{j}\left(t\right)$, j = 1, 2,⋯,J are orthonormal, such that the consecutive scores have decreasing variance. To enable computational solution of FPCA (for details, see [29,30]), it is assumed that both the observations and the eigenfunctions can be approximated by linear combinations of a set of basis functions—for example, of B-splines. The main advantage of FPCA is the fact that it assumes a smooth relationship between the values of the weighting eigenfunctions $\xi_{j}$ at consecutive time points, whereas PCA finds the weights $\xi_{jp}$ for all original random variables independently. This has a good effect of stabilizing the solution and results in a useful interpretation of the eigenfunctions—namely, the values of the first eigenfunction are the biggest in the interval in which the variation between observed functions is the largest, and the consecutive eigenfunctions indicate regions with decreasing variability. Scatterplots of samples at coordinates given by the first two or three FPCA scores are interpreted in the same way as PCA plots. To perform computations, we used the package fda in R based on theory and applications presented in [30].

We applied the FPCA method to individual peaks obtained in the last step of pre-processing, which is the detection of common peaks for all chromatograms.

### 4.5. Peak Quantification

In Method 1, concentrations of compounds in all samples were computed by integrating the chromatograms over peaks obtained after separation. In Method 2, concentrations were represented by values of the first few FPCA scores, with their number determined by the cumulative percentage of explained variation greater than a threshold (we used 80%). In this way, the results were prepared as input data for the statistical analysis.

## 5. Conclusions

In this study, two methods of separating chromatographic peaks were compared. Both FPCA and clustering are appropriate for the analysis of any chromatographic data. Our simulations indicate FPCA as a method that better preserves differences between experimental variants. Real data examples were selected to demonstrate frequent challenges in the interpretation of results in chromatography. The real data examples showed that clustering cannot create clusters corresponding to different peaks when chromatograms are of a similar shape with overlapping peaks in each chromatogram, while FPCA deals with this situation in the same way as with all others. The advantage of clustering is that it provides an explicit definition of the separated peaks. FPCA preserves information about variation in samples, which is crucial when samples are analyzed in different conditions, treatments, etc. We can present each complex peak with a defined number of variables in FPCA. Therefore, both methods have advantages that make them useful for the analysis of large chromatographic datasets.

## Data Availability

Real data examples were selected for 104 identified metabolites submitted to the Metabolights database, identifier: MTBLS52 released 20 November 2014 (available at: http://www.ebi.ac.uk/metabolights/MTBLS52 (accessed on 20 March 2017)).

## Supplementary Materials

The following are available online at https://www.mdpi.com/article/10.3390/metabo11040214/s1, Plant material and instrumental analysis methods [28,29,33,38,47,48,49,50,51,52,53,54].
