# Supplementary material for: Separation of Chromatographic Co-Eluted Compounds by Clustering and by Functional Data Analysis
Source: Metabolites. 2021 Mar 31;11(4):214. doi: 10.3390/metabo11040214 (PMC8065729; doi:10.3390/metabo11040214)
Supplement: Supplementary file 1 [file metabolites-11-00214-s001.pdf]

Supplementary Materials: Plant material and instrumental analysis methods

Plant material cultivation

In the experiment, the following nine spring barley (*Hordeum vulgare* L.) genotypes were used: European varieties Georgia, Maresi, Lubuski, Sebastian, and Stratus; Morex – bred in the USA; and Cam/B1/CI 08887//Cl 0576 (hereinafter referred to as Cam/B1/CI), Harmal, and Maris Dingo/Deir Alla 106 (MDingo) being lines bred in Syria. The experiment was conducted in a greenhouse under partially controlled conditions. Plants were grown in pots filled with loam-sandy soil. The following four water regimes were applied: control – 12% of soil moisture during all vegetation season; drought treatment I – soil moisture in the pots was decreased to 6% when seedlings were at the 3-leaf stage, and this moisture level was achieved within 48 hours and maintained for 10 days; drought treatment II – the decrease in soil moisture to 6% was started when plants were at the stage of the flag leaf and maintained for 14 days; drought treatment I+II – I and II variants of drought applied to the same plants, all details are described in similar experiment in Mikołajczak [47] and Ogrodowicz [48]. The harvested material was immediately frozen in liquid nitrogen and stored at −80°C until metabolite extraction and instrumental analysis.

Leaf samples were harvested from control plants after 1, 6, and 10 days of drought and from the stressed plants after 3, 6, and 10 days; for this presentation, time points 1 and 3 were identified as one point.

Extraction of plant material

All reagents and solvents for extraction and UHPLC/PDA analyzes (methanol, acetonitrile, formic acid, dimethyl sulfoxide, polyamide, and chloroform ultrapure water obtained from a Millipore Direct Q3 device) were from Sigma-Aldrich (Poznań, Poland). Standards of flavonoids (apigenin, apigenin-7-*O*-glucoside, apigenin-6-*C*-glucoside, apigenin-8-*C*-glucoside, isorhamnetin, luteolin, luteolin 7-*O*-glucoside, luteolin 4′-*O*-glucoside, luteolin-8-*C*-glucoside, luteolin-6-*C*-glucoside, kaempferol, kaempferol-3-*O*-glucoside, kaempferol-7-*O*-glucoside, quercetin 3-*O*-galactoside) were purchased from Extrasynthese (Genay, France).

Weighted samples of frozen leaves (about 100 mg) were placed in Eppendorf tubes with 1.4 ml of 80% methanol and the internal standard, homogenized using a ball mill (MM 400, Retsch, Haan, Germany) and subsequently placed in an ultrasonic bath for 30 min and centrifuged (11,000 × *g*) for 10 min. Apigenin (5 μl of 1 mg/ml solution in dimethyl sulfoxide) was used as the internal standard in extracts prepared for the UHPLC analyzes.

Instrumental analysis

Profiling of secondary metabolites was carried out using the Acquity (Waters, Milford, USA) instrument with a photodiode array detector PDAeλ and a BEH C18 column (2.1×150 mm, 1.7 μm particle size) at 40°C with a mobile phase flow rate of 0.4 ml/min. The injection volume was 2 μl. The elution was conducted with water containing 0.1% formic acid (solvent A) and acetonitrile (solvent B). The gradient elution was started at 100% of A and linearly changed to 80% of A in 2 min and then to 70% of A in 8 min and to 5% of A over 1 min, followed by a return to start conditions and re-equilibration for 2 min. UV absorbance was measured in the 230–450 nm wavelength range with a resolution of 2 nm, and data were analyzed with the Empower 2 Chromatography Data Software (Waters). The assumed number of biological replications for experimental combinations was four, but for some samples, the number of replications was smaller than four due to a lack of plant material. For the same reason, data for some time points were missing. As an example in this paper two wavelengths, which are representative for most phenolic compounds, were selected (280 and 330 nm). The retention time range of 2–11 min was

considered. Secondary metabolites were identified using HPLC-MS system according to the previously published approach [29,38,49–51] on the basis of mass spectra, retention times and literature data [52–54]. Then, the signals of compounds identified in the HPLC-MS system were extrapolated to peaks in the UHPLC chromatogram. Several compounds were isolated from barley leaves on a preparative scale and purified using preparative chromatography on a polyamide column followed by preparative HPLC on a reversed phase C18 column. Using ^{1}H, ^{13}C, COSY, NOESY, HMBC, HSQC NMR spectra, structures of the purified compounds were established in [28] and [33]. Data for 104 identified metabolites were submitted to Metabolights database, identification number MTBLS52.
